# Clinical Significance of Pre- and Post-Transplant BAFF Levels in Kidney Transplant Recipients

**DOI:** 10.1371/journal.pone.0162964

**Published:** 2016-09-15

**Authors:** Ji Won Min, Kyoung Woon Kim, Bo-Mi Kim, Kyoung Chan Doh, Min Seok Choi, Bum Soon Choi, Cheol Whee Park, Chul Woo Yang, Yong-Soo Kim, Eun-Jee Oh, Byung Ha Chung

**Affiliations:** 1 Transplant research center, Seoul St. Mary's Hospital, College of Medicine, The Catholic University of Korea, Seoul, Korea; 2 Division of Nephrology, Department of Internal Medicine, Seoul St. Mary's Hospital, College of Medicine, The Catholic University of Korea, Seoul, Korea; 3 Department of Laboratory Medicine, Seoul St. Mary's Hospital, College of Medicine, The Catholic University of Korea, Seoul, Korea; University of Toledo, UNITED STATES

## Abstract

It is well known that pre-transplant B cell activating factor (BAFF) levels are associated with the development of de novo anti-HLA antibodies and antibody mediated rejection post-transplant. However, the clinical significance of BAFF values at allograft rejection has not been determined. In this study, we investigated the clinical significance of pre-transplant BAFF level as well as post-transplant BAFF levels measured when indication biopsy was done. We checked for anti-HLA antibodies in 115 kidney transplant recipients who required allograft biopsy due to an increase in serum creatinine. With the same serum specimen, we measured BAFF levels, and in 78 of these patients, pre-transplant BAFF and anti-HLA antibody levels were detected as well. Patients in each group were divided into tertiles according to BAFF levels. We investigated the relationship between BAFF levels and the occurrence of anti-HLA antibodies. Pre-transplant BAFF levels showed significant association with pre-transplant sensitization, and also with early rejection (Tertile 3, 26.9% vs. Tertile 1, 11.5%; *P*<0.05). Post-transplant BAFF levels showed significant association with pre-transplant sensitization, but did not show association with anti-HLA antibodies and positive donor-specific antibodies at the time of biopsy. We did not find any association between post-transplant BAFF levels and allograft biopsy results, Banff scores and microvascular inflammation scores. In conclusion, pre-transplant BAFF levels are associated with pre-transplant sensitization and are useful in predicting allograft rejection. But post-transplant BAFF levels measured at the time of indication biopsy are not associated with the appearance of de novo HLA-DSA, allograft rejection, biopsy findings and other allograft outcomes.

## Introduction

The role of B-cells in kidney transplantation, especially in acute and chronic antibody-mediated rejection (AMR) and transplantation tolerance, has been repeatedly emphasized in recent studies [1,2]. Besides the well-known effects on humoral immunity, B cells also modulate the function of T cells by antigen presentation, co-stimulation, and cytokine secretion [3]. Therefore, the monitoring of cytokines or chemokines associated with B cell activation or survival has been proposed as an important strategy to predict allograft outcome [1,2].

B-cell activating factor (BAFF) is a cytokine belonging to the tumor necrosis factor family that plays a role in the survival, proliferation and differentiation of B-cells [4,5]. Several studies have already demonstrated the correlation of elevated BAFF levels with chronic graft versus host disease [6] and autoimmune disease such as systemic lupus erythematosus (SLE), Sjogren disease, and rheumatoid arthritis [7,8]. In kidney transplantation, the clinical significance of high levels of BAFF has also been sporadically reported and most of them focused on the clinical significance of pre-transplant BAFF levels in the prediction of post-transplant clinical outcomes [9–11]. For example, pre-transplant soluble BAFF levels showed correlation with the de novo appearance of donor-specific antigens (DSA) [12], while another report showed that pre-transplant BAFF levels were associated with increased AMR and decreased rejection free survival [13]. The above data strongly suggest the effects of pre-transplant BAFF levels on the outcomes of kidney transplantation (KT).

However, the clinical significance of monitoring of post-transplant BAFF levels has not yet been determined. Therefore in this study, we measured both pre-transplant and post-transplant BAFF serum levels in kidney transplant recipients, with the intention to investigate the significance of pre and post-transplant BAFF levels in predicting clinical outcomes such as acute rejection and allograft failure.

## Materials and Methods

### Study population

We analyzed a hundred and thirty cases of serum taken from 115 patients at the time of indication biopsy performed between January, 2010 and December, 2014 at Seoul St. Mary’s Hospital. Pre-transplant serum taken just before initiation of any immune suppressants or desensitization therapy such as rituximab or plasmapheresis was available in 78 of these cases. We measured serum BAFF level in the 130 post-transplant serums and also in the 78 pre-transplant serums and investigated the clinical significance of both. This study received approval of the Institutional Review Board of Seoul St. Mary’s Hospital (KC13TNMI0701) and all patients signed an informed consent before inclusion.

### Indication biopsy procedure

In our center, indication biopsy was done in patients with an increase in serum creatinine of 20% above baseline value. The biopsy was performed as previously described [14], using a 16-gauge biopsy gun with ultrasonic localization. Histopathological diagnosis was made based on the revised Banff working classification [15]. C4d deposition was detected using monoclonal antibodies (Biogenesis, Poole, U.K.; dilution 1:50) for indirect immunofluorescence (IF) staining. Microvascular inflammation (MVI) score was defined as the sum of the glomerulitis (g) and peritubular capillaritis (ptc) scores.

### Detection of donor specific anti-HLA antibody

HLA antibodies were detected using Lifecodes LSA Class I and Class II kits (Gen-Probe Transplant Diagnostic Inc., Stamford, CT, USA) or LABScreen Single Antigen (One Lambda Inc., A Thermo Fisher Scientific Brand, Canoga Park, CA), according to the manufacturer’s instructions. Fluorescence was analyzed using the Quick-Type User’s Manual Research Use Only program, version 2.4, of the LABScan100 flow cytometer (Luminex Corp, Austin, TX). Normalized median fluorescence intensity (MFI) greater than 1,000 were considered positive. HLA typing was done by DNA molecular typing in all donors and patients using sequence-specific oligonucleotide probes (SSOP) with LIFECODES HLA SSO typing kits (Immucor, Stamford, CT, USA). HLA-DSA was defined as the anti-HLA antibodies of the recipient corresponding with HLA types of the donor.

### Measurement of serum BAFF level

Serum BAFF levels were measured using serum that had been stored at -70°C. Commercially available ELISA kit (R&D Systems, Minneapolis, MN, USA) was used according to the manufacturer’s recommendations and results were expressed in pg/mL.

### Comparison of clinical outcome parameters

Correlation of serum BAFF levels with pre-transplant sensitization status such as PRA levels, and detection of HLA-DSA were observed, as well as the development of biopsy proven acute rejection (antibody-mediated and T-cell mediated), and allograft survival post-transplant. Patients in both the pre-transplant (n = 78) and post-transplant groups (n = 130) were also divided into tertile groups (tertile 1, 2 and 3) according to BAFF levels, and clinical parameters or outcomes were compared across the 3 BAFF tertiles. Detailed pathologic findings based on the Banff classification were compared among tertiles in the post-transplant BAFF measured group. In 78 patients in whom both pre- and post-transplant BAFF level were measured, we calculated the change of BAFF level after KT in comparison with before KT (delta BAFF) in each patient and observed the correlation of these values with the above mentioned parameters. These patients were also divided into three tertiles according to delta BAFF value and clinical parameters or outcomes were compared.

### Statistical analysis

Results are described as mean ± standard deviation (SD) or median and range for continuous variables and as percentages for categorical data. Comparisons were made with chi-square test for categorical data and Student’s t-test for continuous variables. Allograft survival was compared between tertiles by Kaplan-Meier analysis with a log-rank test. Statistical analysis was performed using Statistical Package for the Social Sciences (SPSS) software (version 20 for Windows, SPSS Inc., Chicago, IL) and the statistical package MedCalc version 15.5 (MedCalc, Mariakerke, Belgium). Results with P values below 0.05 were considered significant.

## Results

### Comparison of baseline and clinical characteristics according to pre- and post-transplant BAFF levels

Post-transplant BAFF levels were measured concomitantly with 130 indication biopsies in 115 patients and median post-transplant BAFF level was 99.49 pg/mL (9.63~897.89 pg/mL). In 78 patients in whom pre-transplant serum was available, median pre-transplant BAFF level was 7.48 pg/mL (range of 0.91~ 60.97 pg/mL). Patients were divided into 3 tertile groups according to serum BAFF levels and a comparison of baseline and clinical characteristics among the tertiles were made (Tables 1 and 2). There was no significant difference in the baseline characteristics such as age, gender, re-transplantation number, HLA mismatch number or immunosuppressive drugs among the tertiles in both the pre- and post-transplant sample groups. However, the percentage of patients who underwent pre-transplant desensitization therapy was significantly higher in tertile 3 compared to the other tertiles (55.8% in tertile 3 vs. 13.6% in tertile 2 vs. 7.1% in tertile 1, P < 0.05) in the post-transplant patient group.

**Table 1:** Clinical characteristics of pre-transplant BAFF-measured patients.

N = 78		Tertile 1 (n = 26)	Tertile 2 (n = 26)	Tertile 3 (n = 26)	P-value
**Age (year)**		45.4 ± 9.6	46.2 ± 16.4	43.5 ± 11.4	0.947
**Gender; male, n (%)**		17 (65.4)	13 (50)	16 (61.5)	0.110
**Retransplantation, n (%)**		1 (3.8)	4 (15.4)	3 (11.5)	0.569
**HLA mismatch number**		3.4 ± 1.6	3.4 ± 1.7	3.1 ± 1.4	0.382
**Deceased donor, n (%),**		6 (23.1)	8 (30.8)	2 (7.7)	0.621
**Primary renal disease**					0.933
	Chronic GN, n (%)	7 (26.9)	11 (42.3)	10 (38.5)	
	DM, n (%)	6 (23.1)	2 (7.7)	4 (15.4)	
	HTN, n (%)	6 (23.1)	3 (11.5)	2 (7.7)	
	ADPKD, n (%)	2 (7.7)	1 (3.8)	3 (11.5)	
	Others, n (%)	5 (19.2)	9 (34.6)	7 (26.9)	

BAFF, B cell activating factor; HLA, human leukocyte antigen; GN, glomerulonephritis; DM, diabetes mellitus; HTN, hypertension; ADPKD, autosomal dominant polycystic kidney disease.

**Table 2:** Clinical characteristics of post-transplant BAFF-measured patients.

N = 115		Tertile 1 (n = 43)	Tertile 2 (n = 44)	Tertile 3 (n = 43)	P-value
**Follow-up (month)**		11.9 ± 15.9	4.9 ± 6.9	5.5 ± 5.7	0.151
**Age (year)**		45.2 ± 10.5	43.7 ± 12.3	44.3 ± 9.4	0.947
**Gender; male, n (%)**		32 (74.4)	26 (59.1)	23 (53.5)	0.110
**Retransplantation, n (%)**		2 (4.7)	5 (11.4)	5 (11.6)	0.554
**HLA mismatch number**		3.6 ± 1.3	3.5 ± 1.7	3.4 ± 1.06	0.427
**Deceased donor, n (%),**		14 (32.6)	11 (25.0)	10 (23.3)	0.424
**Pre-transplant desensitization, n (%)**		3 (7.0)	6 (13.6)	24 (55.8)	<0.001
**Main immunosuppressant, n (%)**					0.428
	Cyclosporine	3 (7.0)	1 (2.3)	1 (2.3)	
	Tacrolimus	37 (86.0)	41 (93.2)	38 (88.4)	

BAFF, B cell activating factor; HLA, human leukocyte antigen

### Association of pre-transplant BAFF levels with pre-sensitization and post-transplant allograft rejection

**Fig 1:**
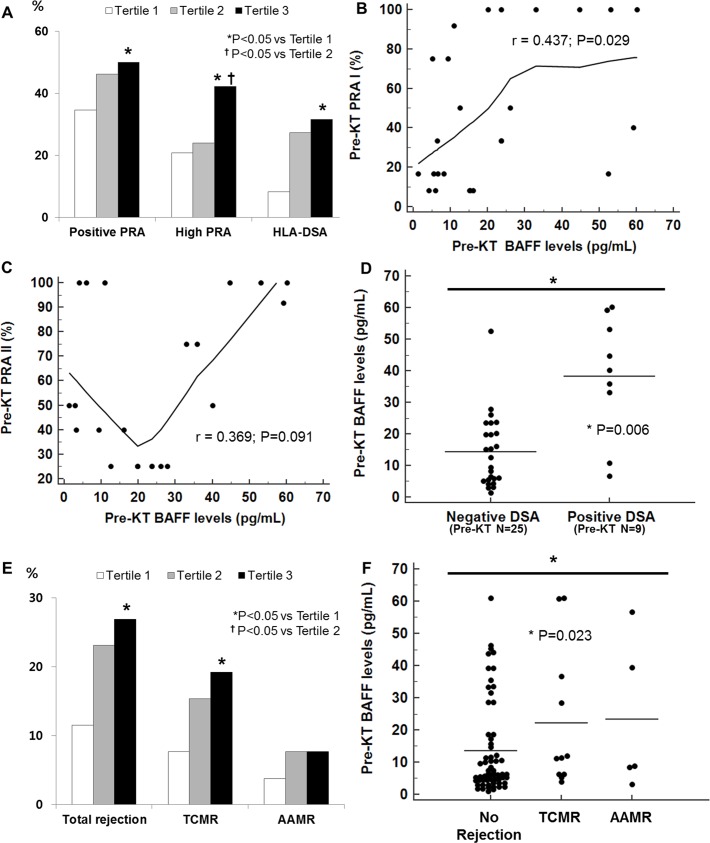
Association of pre-transplant BAFF with various clinical parameters. Comparison of (A) pre-sensitization among pre-transplant BAFF tertiles, and (B) individual pre-transplant BAFF levels with pre-transplant PRA I, (C) PRA II, and (D) presence of pre-transplant HLA-DSA, showed that elevated serum BAFF levels before transplantation were significantly associated with pre-transplant sensitization. Comparison of (E) total acute rejection, TCMR and AAMR among pre-transplant BAFF tertiles and (F) individual pre-transplant BAFF levels with the presence of rejection showed that pre-transplant BAFF levels were associated with early rejection. BAFF, B cell activating factor; PRA, panel reactive antibody; HLA-DSA, anti-HLA donor specific antibody; KT, kidney transplant; TCMR, T cell mediated rejection; AAMR, acute antibody mediated rejection.

We observed the correlation of serum BAFF levels with pre-transplant sensitization status and early acute rejection episodes, defined as rejection within 6 months from KT, and also compared the parameters among the 3 tertile groups. Higher serum BAFF levels before transplantation were significantly associated with pre-transplant sensitization; 50% of patients in tertile 3 showed positive PRA compared with 34.6% of patients in tertile 1 (P = <0.05), 42.3% of patients in tertile 3 showed high PRA levels (defined as PRA levels higher than 50%) compared with 20.8% of patients in tertile 1 (P = <0.05) (Fig 1A). Higher pre-transplant PRA I levels were associated pre-tranplant BAFF levels (r = 0.437, P = 0.029) (Fig 1B) but PRA II levels did not show association (r = 0.369, P = 0.091) (Fig 1C). Presence of HLA-DSA was noted in 31.6% of patients in tertile 3 compared with 8.3% of patients in tertile 1(P = <0.05) (Fig 1A) while significantly higher BAFF levels were noted in patients with positive DSA pre-transplant (mean±SD; negative DSA group 14.33±11.54 pg/mL; positive DSA group 38.22±19.27 pg/mL; P = 0.006) (Fig 1D). BAFF levels failed to show correlation with MFI titers however (r = -0.203; P = 0.574) (S1A Fig). After transplantation, higher pre-transplant BAFF levels also showed positive association with early rejection. In tertile 3, the proportion of patients who suffered acute rejection was 26.9%, but in tertile 1, it was only 11.5% (P = <0.05) (Fig 1E). Higher levels of pre-transplant BAFF were observed in patients with TCMR and even higher levels in those with acute AMR (AAMR) compared to patients with no rejection (mean±SD; no rejection group 13.58±14.47 pg/mL; TCMR group 22.12±21.69 pg/mL; AAMR group 23.29±23.53 pg/mL; P = 0.023) (Fig 1F).

### Association between post-transplant BAFF levels and pre-sensitization, development of allograft rejection, and allograft survival rate

**Fig 2:**
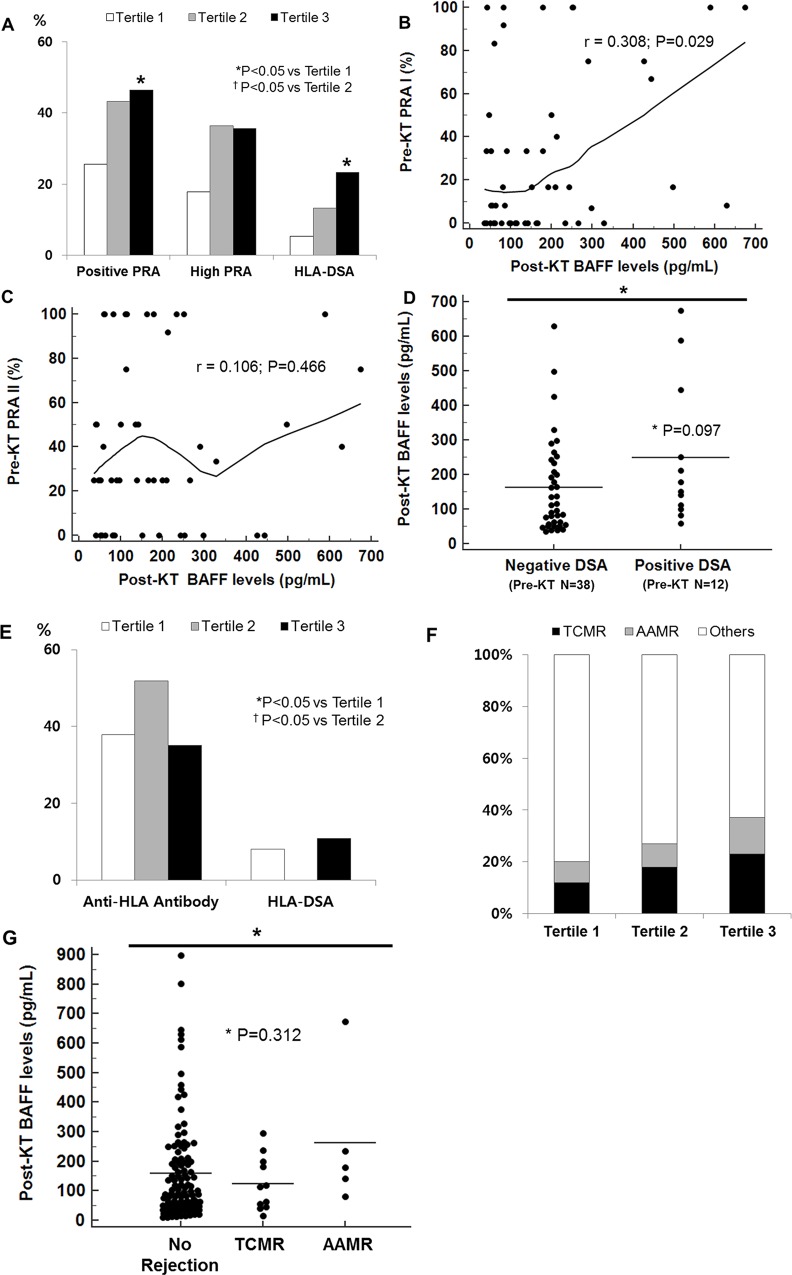
Association of post-transplant BAFF with various clinical parameters. Comparison of (A) pre-sensitization among post-transplant BAFF tertiles, and (B) individual post-transplant BAFF levels with pre-transplant PRA I, (C) PRA II, and (D) presence of pre-transplant HLA-DSA, showed that elevated serum BAFF levels after transplantation were associated with pre-transplant sensitization. Comparison of (E) the prevalence of post-transplant anti-HLA antibody and HLA-DSA, (F) TCMR, AAMR and no rejection, among post-transplant BAFF tertiles, and (G) individual pre-transplant BAFF levels with the presence of rejection, showed that post-transplant BAFF levels were not associated with rejection. BAFF, B cell activating factor; PRA, panel reactive antibody; HLA-DSA, anti-HLA donor specific antibody; KT, kidney transplant; TCMR, T cell mediated rejection; AAMR, acute antibody mediated rejection.

**Fig 3:**
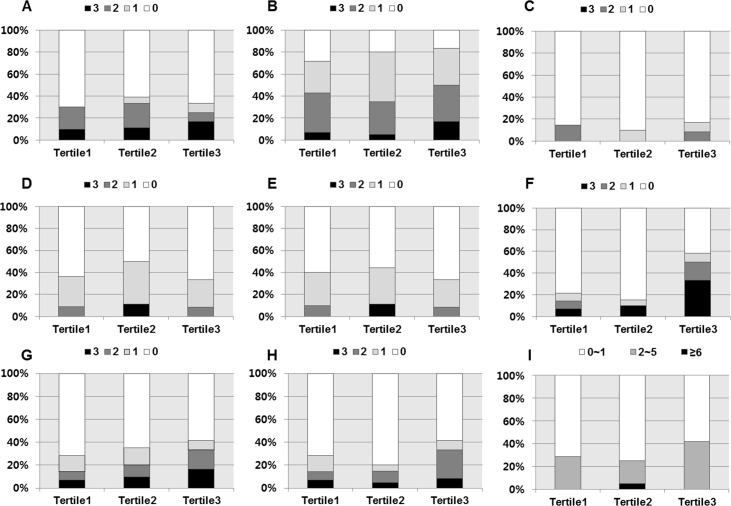
Association of post-transplant BAFF levels with Banff score. Pathological findings based on the Banff classification, (A) t score (B) i score (C) v score (D) ct score (E) ci score (F) c4d score (G) g score (H) ptc score and (I) MVI scores, did not show significant association with post-transplant BAFF levels. BAFF, B cell activating factor; MVI, microvascular inflammation.

**Fig 4:**
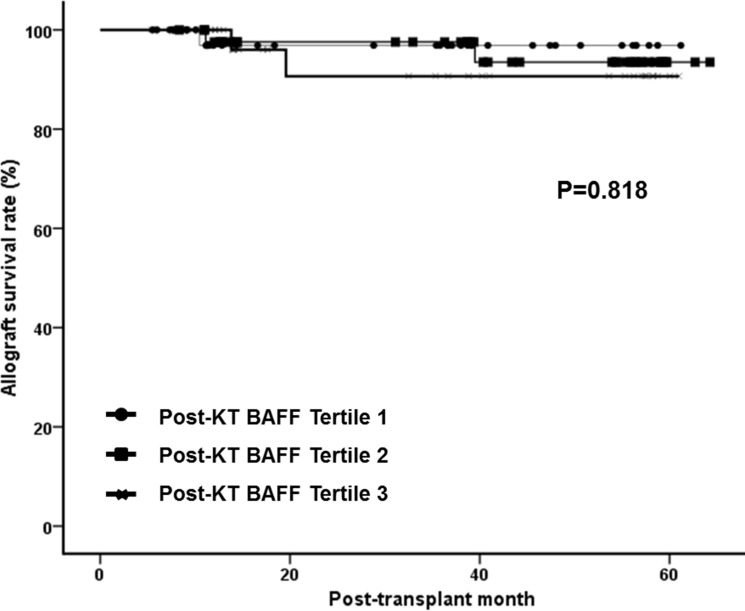
Impact of post-transplant BAFF levels on allograft outcomes. Note that post-transplant BAFF levels did not show significant correlation with allograft survival rate after allograft biopsy. KT, Kidney transplantation; BAFF, B cell activating factor.

Post-transplant BAFF levels showed significant association with pre-transplant sensitization; 46.5% of patients in tertile 3 showed positive PRA, compared with 25.6% of patients in tertile1(P = <0.05) and presence of HLA-DSA was found in 23.3% of patients in tertile 3 while only 5.5% of tertile 1 patients showed positive HLA-DSA before transplantation (P = <0.05) (Fig 2A). PRA I levels showed weak but significant correlation with post-transplant BAFF levels (r = 0.308, P = 0.029) (Fig 2B), while PRA II did not show correlation (r = 0.106, P = 0.466) (Fig 2C). Patients with positive HLA-DSA showed tendency toward higher mean levels of post-transplant BAFF (mean±SD; negative DSA group 162.44±136.88 pg/mL; positive DSA group 249.60±205.96 pg/mL; P = 0.097) (Fig 2D). However, post-transplant BAFF levels did not show association with anti-HLA antibodies and positive donor-specific antibodies at the time of indication biopsy (Fig 2E). We also did not find any association between post-transplant BAFF levels and allograft biopsy results (Table 3 and Fig 2F and 2G). Pathological findings based on the Banff classification did not show significant association with post-transplant BAFF levels (Fig 3A–3H). The microvascular inflammation score calculated by adding the g score and ptc score did not show correlation with higher BAFF levels (Fig 3I). As indicated in Fig 4, post-transplant BAFF levels did not show significant correlation with allograft survival rate after allograft biopsy.

**Table 3:** Comparison of allograft biopsy findings in the post-transplant BAFF measured group.

	Tertile 1 (n = 43)	Tertile 2 (n = 44)	Tertile 3 (n = 43)	*P*
**TCMR, n (%)**	10 (23.3)	8 (18.2)	10 (23.3)	0.550
**Acute or chronic AMR, n (%)**	3 (7.0)	4 (9.1)	5 (11.6)	0.550
**Mixed rejection, n (%)**	9 (20.1)	7 (15.9)	2 (4.7)	0.550
**CNI toxicity, n (%)**	2 (4.7)	2 (4.5)	1 (2.3)	0.550
**BKVAN, n (%)**	0 (0)	0 (0)	1 (2.3)	0.550
**Others, n (%)**	19 (44.2)	23 (52.3)	24 (55.8)	0.550

BAFF, B cell activating factor; TCMR, T cell mediated rejection; AMR, antibody mediated rejection; CNI, calcineurin inhibitor; BKVAN, BK virus associated nephropathy.

### Association between delta BAFF levels and pre-sensitization, post-transplant detection of HLA-DSA and development of acute rejection

**Fig 5:**
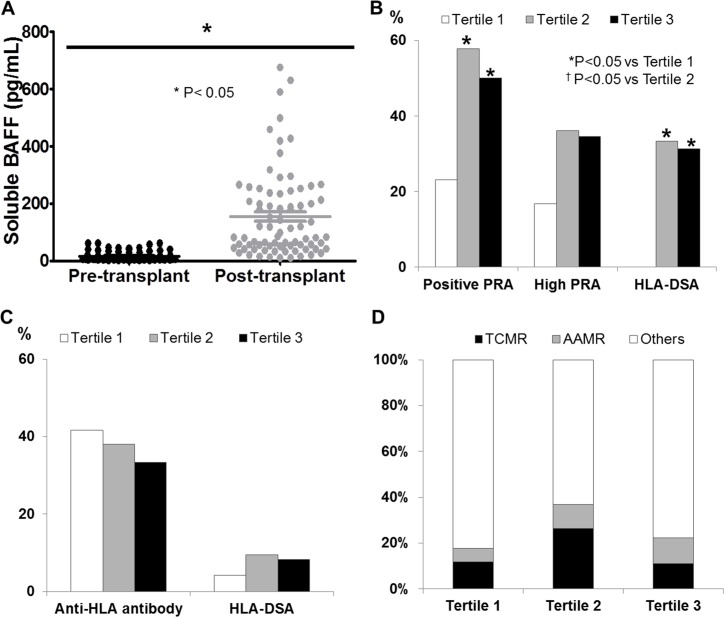
Association of delta BAFF tertiles with various clinical parameters. (A) Post-transplant serum BAFF levels were significantly higher than pre-transplant levels. Comparison of (B) pre-sensitization, (C) the prevalence of post-transplant anti-HLA antibody and HLA-DSA, and (D) TCMR, AAMR and no rejection among delta BAFF tertiles, showed that delta BAFF levels were significantly associated with pre-transplant sensitization but not with the prevalence of post-transplant anti-HLA antibodies, HLA-DSA or allograft biopsy findings. BAFF, B cell activating factor; PRA, panel reactive antibody; HLA-DSA, anti-HLA donor specific antibody; TCMR, T cell mediated rejection; AAMR, acute antibody mediated rejection.

In 78 patients, in whom both pre and post BAFF level were available, we compared the change of serum BAFF levels before and after transplantation. Serum BAFF levels showed significant increase at post-transplant compared to pre-transplant (P<0.05) (Fig 5A). Median delta BAFF level was 75.15 pg/mL (range of -13.85~671.03 pg/mL). Delta BAFF levels showed significant association with pre-transplant sensitization, represented by positive PRA (Tertile 3, 50% vs. Tertile 1, 23.1%; Tertile 2, 57.7% vs. Tertile 1, 23.1%; P = <0.05), high PRA (Tertile 3, 34.6% vs. Tertile 1, 16.7%; P = <0.05), and presence of HLA-DSA before transplantation (Tertile 3, 31.2% vs. Tertile 1, 0%; P = <0.05) (Fig 5B). However, delta BAFF levels did not show association with the presence of anti-HLA antibodies and positive donor-specific antibodies at the time of biopsy (Fig 5C) and did not show association with allograft rejection according to biopsy results (Fig 5D). Individual delta BAFF levels failed to show significant correlation with the different parameters except for a weak correlation with PRA I (S2A–S2D Fig).

## Discussion

In this study, we sought to analyze the clinical significance of measuring pre-transplant and post-transplant serum BAFF levels in kidney transplant recipients We found that although pre-transplant BAFF was associated with pre-sensitization and allograft rejection as in previous studies [12,13], post-transplant BAFF correlated only with pre-sensitization and not with appearance of de novo HLA-DSA, allograft rejection, biopsy findings and other allograft outcomes.

To determine the clinical significance of serum BAFF levels measured before and after transplantation, we chose to observe the correlation of BAFF levels with clinical parameters, and then to divide each group of patients into tertiles according to serum BAFF levels as there has been no reference range yet that has been agreed upon, and levels vary enormously from study to study [13,16,17]. On comparing the pre-sensitization status of patients with different serum BAFF levels, we excluded patients with zero PRA levels as the BAFF levels in these patients may be related to other antibodies besides anti-HLA antibodies [18]. Our results show that higher pre-transplant BAFF levels are associated with higher prevalence of pre-transplant sensitization and increased early allograft rejection. This result is in correlation with previous studies which suggests that BAFF can activate naive B cells, resulting in pre-transplant HLA immunization and an increase in the risk for allograft rejection [9,11–13]. Interestingly, serum BAFF levels were associated with the amount of antibodies demonstrated by PRA values but not with the strength of the HLA-DSA immunoglobulin as with previous studies [13].

However, in the present study, we were more interested in determining the clinical significance of measuring post-transplant BAFF levels, as this aspect has not been thoroughly investigated yet. We measured post-transplant serum BAFF levels in samples taken at the time of indication biopsy in hopes to investigate the relationship between BAFF levels and allograft biopsy results. Interestingly, we found that post-transplant serum BAFF levels also showed significant association with pre-transplant sensitization. The reason is unclear but, it is possible that once humoral immunity has been activated before transplantation, high BAFF levels may persist until the post-transplant period. This may explain the high risk of chronic antibody mediated rejection and allograft dysfunction during long-term follow up especially in patients who were highly sensitized pre-transplant [19,20].

Contrary to our expectations, no significant associations between post-transplant BAFF levels and allograft biopsy findings regarding rejection, findings based on the Banff classification and allograft survival, were found. Our negative findings may be explained by previous studies of serum BAFF kinetics in renal transplantation and chronic GVHD [6,17]. These studies show how serum BAFF levels increase in the year following transplantation, due to several factors such as compensatory effects of B-cell depleting therapies, protocol tapering of immunosuppressants and weak alloresponses stimulated by the graft. This peaking of serum BAFF happens regardless of antibody status; DSA, non-DSA, and HLA-negative groups did not show significant difference.

To exclude the effect of B-cell depleting therapies, we performed a subgroup analysis excluding patients who underwent Rituximab desensitization therapy. According to our center’s desensitization protocols, pre-sensitized patients with high PRA or positive HLA-DSA are treated with rituximab (375 mg/m^2^, intravenously) a month before transplantation. B-cell depleting agents such as this have been reported to be associated with significant elevation of serum BAFF levels for more than 1 year after transplantation [16,21,22]. Comparison of post-transplant serum BAFF levels in patients who underwent Rituximab therapy and those who did not, showed that post-transplant BAFF levels were significantly higher in patients who underwent Rituximab therapy (median 251.48 pg/mL vs. 80.73 pg/mL in patients without Rituximab therapy, *P* = 0.000) (S3A Fig). However, analysis of the subgroup divided into tertiles according post-transplant serum BAFF levels showed significant association of post-transplant BAFF levels and pre-transplant sensitization, but no association with the presence of HLA antibody or HLA-DSA (S3B and S3C Fig). These results proved to be consistent with results of the whole group analysis in this study.

Finally, we performed an additional analysis to investigate the change in BAFF levels between pre and post-transplantation. We also evaluated whether delta BAFF levels were associated with post-transplant clinical outcomes in 78 patients in whom pre and post-transplant serum were available. Delta BAFF levels also showed significant association with pre-transplant sensitization but not with post-transplant DSA or allograft rejection. This non-specific rise in serum BAFF levels immediately after transplantation, may similarly be explained by the activation of various BAFF-producing immune cells due to normal immune responses stimulated by the transplanted allograft [23].

There are a few limitations to this study. As mentioned earlier, we only took post-transplant samples at the time of indication biopsy, therefore we could not assess the point at which serum BAFF levels may have shown correlation with allograft dysfunction or rejection. A longitudinal study with sampling at multiple time points will be needed to determine whether post-transplant serum BAFF is at any time useful in predicting graft outcomes. Secondly, in our study, we were only able to measure serum BAFF levels which represent a fraction of the total BAFF pool. We could additionally analyze the cell-membrane-bound form of BAFF by measuring the BAFF mRNA on peripheral blood mononuclear cells as previously done by Thibault-Espitia et al [12].

In conclusion, this is the first study to examine the clinical significance of both pre and post-transplant serum BAFF levels in adult kidney transplant recipients. Pre-transplant BAFF levels may be useful in predicting allograft rejection, but post-transplant BAFF levels measured at the time of renal dysfunction failed to show significant correlation with allograft outcomes.

## Supporting Information

S1 Fig **(A) Correlation of pre-transplant BAFF levels with DSA titer.** Note that no significant correlation was observed. BAFF, B cell activating factor; KT, kidney transplant; DSA, donor specific antibody; MFI, median fluorescence intensity.
(TIF)

S2 Fig Association of delta BAFF levels with various clinical parameters. Comparison of delta BAFF levels with (A) pre-transplant PRA I, (B) PRA II, (C) presence of pre-transplant HLA-DSA, and (D) post-transplant acute rejection showed weak association with pre-transplant PRA I but did not show significant association with PRA II, prevalence of pre-transplant HLA-DSA or allograft biopsy findings. BAFF, B cell activating factor; PRA, panel reactive antibody; DSA, donor specific antibody; TCMR, T cell mediated rejection; AAMR, acute antibody mediated rejection.
(TIF)

S3 Fig Subgroup analysis excluding patients who underwent Rituximab desensitization therapy. (A) Comparison of post-transplant BAFF levels in patients who underwent Rituximab desensitization therapy and those who did not showed that serum BAFF levels were significantly higher in the group that underwent Rituximab desensitization therapy. Comparison of (B) pre-transplant sensitization and (C) prevalence of post-transplant HLA-DSA among post-transplant BAFF tertiles in the subgroup analysis showed that post-transplant BAFF levels in the subgroup of patients excluding those who underwent desensitization therapy were also significantly associated with pre-transplant sensitization but not with the prevalence of post-transplant anti-HLA antibody and HLA-DSA. BAFF, B cell activating factor; PRA, panel reactive antibody; HLA-DSA, anti-HLA donor specific antibody.
(TIF)
