# Supplementary figures and images for: Clinical Significance of Pre- and Post-Transplant BAFF Levels in Kidney Transplant Recipients
Source: PLoS One. 2016 Sep 15;11(9):e0162964. doi: 10.1371/journal.pone.0162964 (PMC5025051; doi:10.1371/journal.pone.0162964)

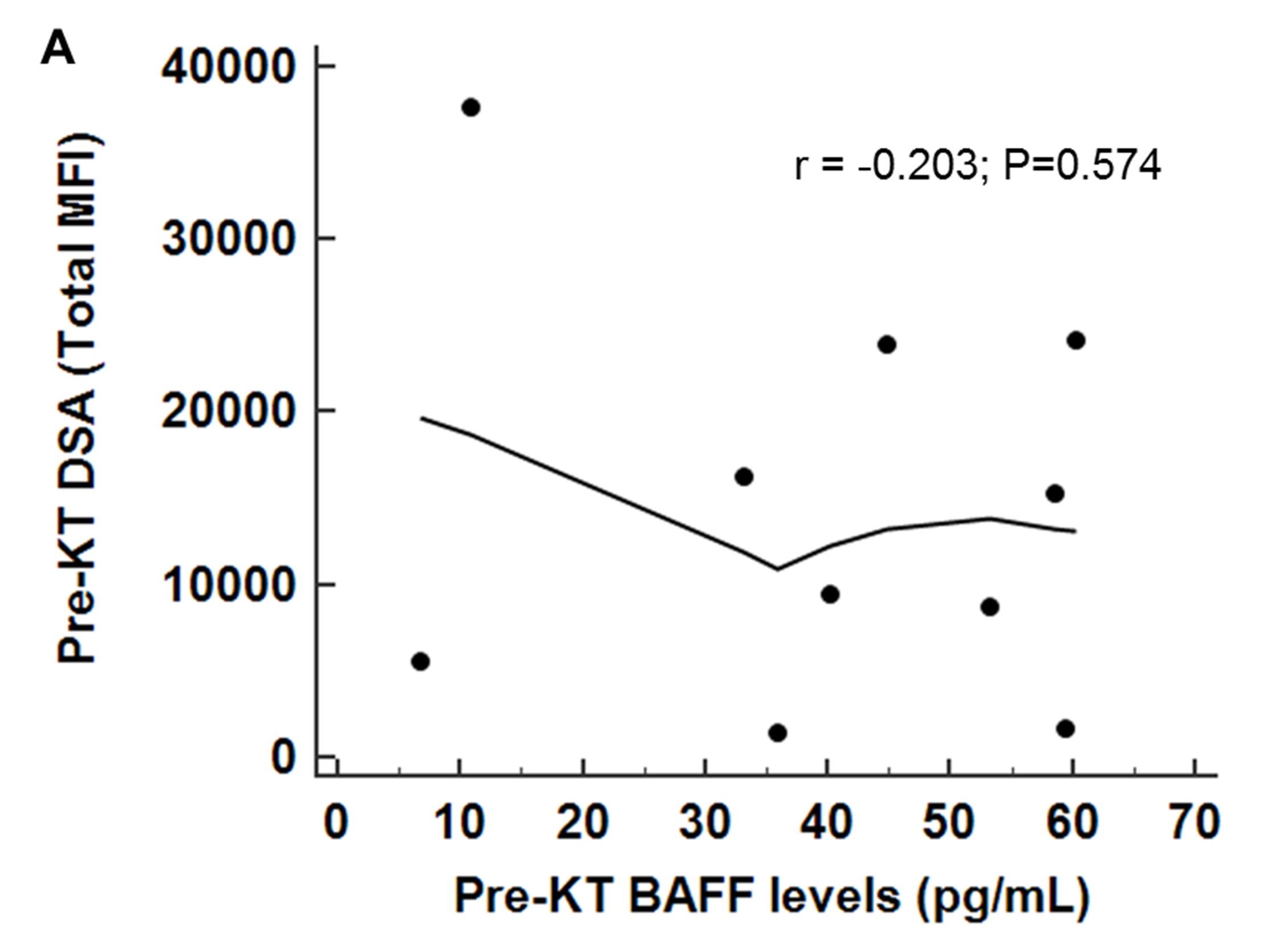

Supplement: S1 Fig — (A) Correlation of pre-transplant BAFF levels with DSA titer. Note that no significant correlation was observed. BAFF, B cell activating factor; KT, kidney transplant; DSA, donor specific antibody; MFI, median fluorescence intensity. (TIF) [file pone.0162964.s001.tif]

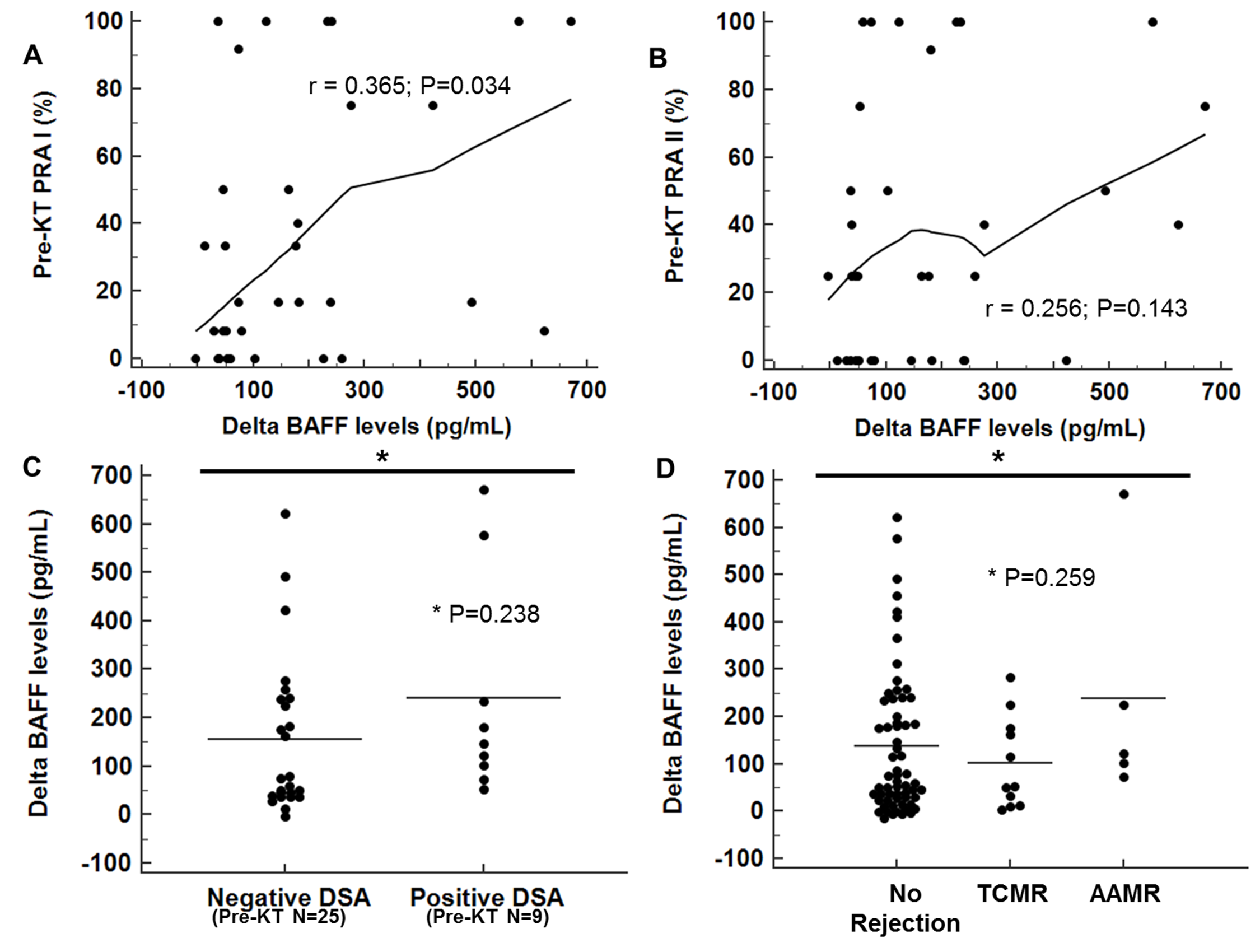

Supplement: S2 Fig — Comparison of delta BAFF levels with (A) pre-transplant PRA I, (B) PRA II, (C) presence of pre-transplant HLA-DSA, and (D) post-transplant acute rejection showed weak association with pre-transplant PRA I but did not show significant association with PRA II, prevalence of pre-transplant HLA-DSA or allograft biopsy findings. BAFF, B cell activating factor; PRA, panel reactive antibody; DSA, donor specific antibody; TCMR, T cell mediated rejection; AAMR, acute antibody mediated rejection. (TIF) [file pone.0162964.s002.tif]

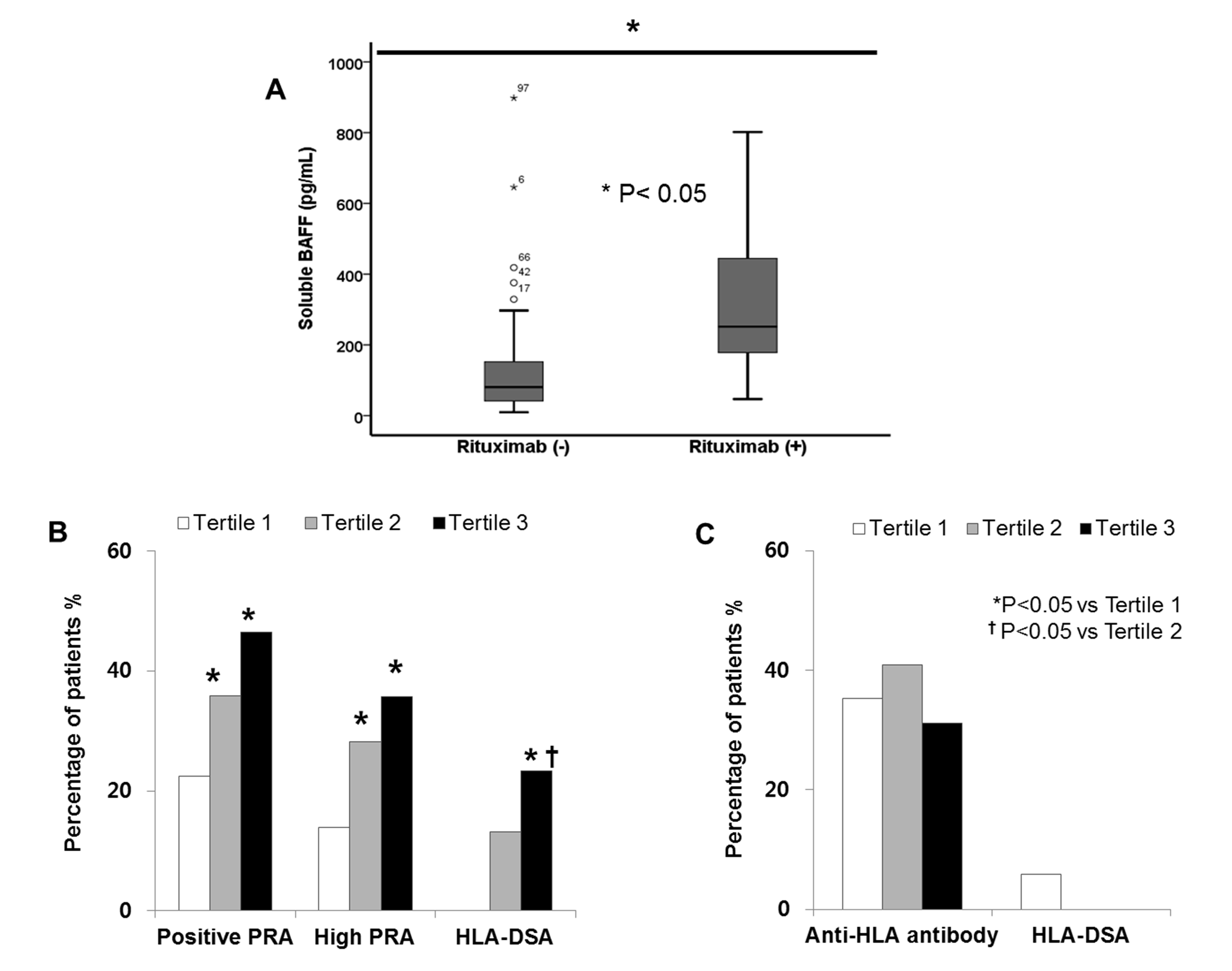

Supplement: S3 Fig — (A) Comparison of post-transplant BAFF levels in patients who underwent Rituximab desensitization therapy and those who did not showed that serum BAFF levels were significantly higher in the group that underwent Rituximab desensitization therapy. Comparison of (B) pre-transplant sensitization and (C) prevalence of post-transplant HLA-DSA among post-transplant BAFF tertiles in the subgroup analysis showed that post-transplant BAFF levels in the subgroup of patients excluding those who underwent desensitization therapy were also significantly associated with pre-transplant sensitization but not with the prevalence of post-transplant anti-HLA antibody and HLA-DSA. BAFF, B cell activating factor; PRA, panel reactive antibody; HLA-DSA, anti-HLA donor specific antibody. (TIF) [file pone.0162964.s003.tif]
